# High resolution 3D diffusion MRI of carotid plaque on 3T

**DOI:** 10.1186/1532-429X-16-S1-O28

**Published:** 2014-01-16

**Authors:** Yibin Xie, Wei Yu, Zhaoyang Fan, Christopher T Nguyen, Jing An, Zhaoqi Zhang, Debiao Li

**Affiliations:** 1 Biomedical Imaging Research Institute, Cedars Sinai Medical Center, Los Angeles, California, USA; 2 Bioengineering, University of California, Los Angeles, Los Angeles, California, USA; 3 Anzhen Hospital, Beijing, China; 4 Siemens Healthcare, Beijing, China

## Background

Diffusion MRI (DWI and ADC) has been shown to provide excellent contrast for characterizing carotid plaque components such as necrotic lipid core and intraplaque hemorrhage ex vivo [1] and in vivo [2]. However, conventional 2D EPI-based methods have suboptimal image quality and limited spatial resolution leading to difficulties in ADC calculation, limiting the clinical utility of this technique. The purpose of this work is to develop a new diffusion imaging method for carotid plaque characterization that (1) allows 3D imaging; (2) improves spatial resolution (0.6 × 0.6 × 2 mm3); and (3) provides consistent and high image quality.

## Methods

Diffusion weighting was introduced with a separate diffusion preparation module from readout, allowing multi-shot high resolution 3D imaging. Readout was designed based on 3D TSE for good and consistent image quality at 3T. Reduced field-of-view with inner-volume refocusing was developed to reduce TSE scan time by 67%. Bipolar diffusion sensitizing gradients were used to compensate first-order motion and minimize eddy currents. Arterial blood was suppressed with DIR for better vessel wall delineation. Six healthy and two atherosclerosis subjects were scanned on a 3T scanner (Siemens Verio) with the following parameters: pulse-triggered; 3D transverse slab with in-plane resolution = 0.6 × 0.6 mm2; slice thickness = 2 mm; b = 30 and 300 s/mm2 along the slice direction; total scan time = 5.5' ± 0.6'.

## Results

**Figure 1:**
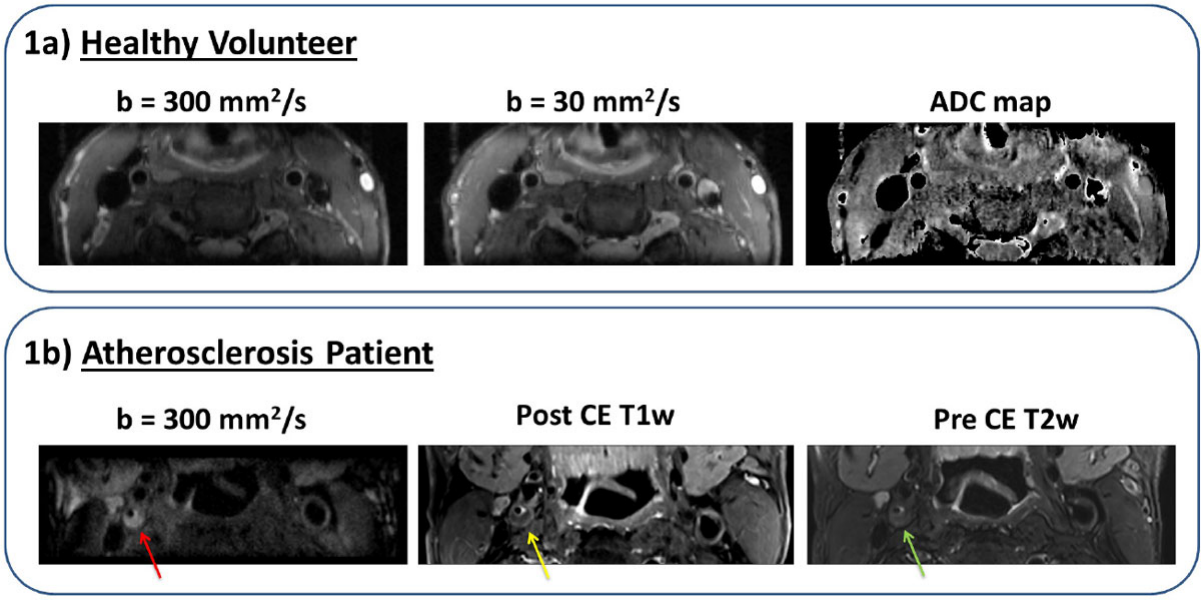
**a (healthy subject): Diffusion weighted image (DWI) at low b-value (30 mm2/s), high b-value (300 mm2/s) and calculated ADC map**. The vessel walls are clearly visible with blood suppression. 1b (atherosclerosis patient): DWI at high b-value compared with post-contrast T1 weighted image and pre-contrast T2 weighted image. Note that part of the plaque area (pointed by arrow) is hyper-intense in DWI signifying lower diffusion in that area. The same area is hypo-intense in the post-contrast T1 weighted image suggesting lipid-rich necrotic core.

**Figure 2:**
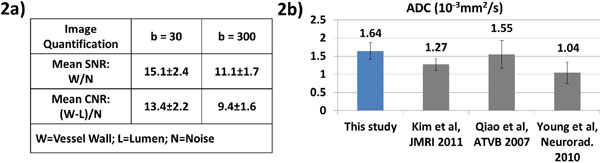
**a: Vessel wall SNR, CNR at both b-values measured respectively by region-of-interest analysis (vessel wall, lumen, remote area)**. 2b: Apparent diffusion coefficient (ADC) meansurement of healthy carotid vessel wall compared with previous in vivo and ex vivo studies.

High resolution DWI of carotid wall with excellent image quality has been achieved at 3T on both healthy and atherosclerosis subjects. Images at both b-values and ADC map showed clear delineation of vessel wall with successful motion compensation (Figure 1a). Quantification by region-of-interest analysis showed satisfactory mean SNR of 15.1 and 11.1 for the vessel wall at the two b-values, respectively. Effective blood suppression throughout the slices resulted wall-to-lumen CNR in the range of 9.4 to 13.4 (Figure 2a). Healthy arterial wall ADC value averaged 1.64 ± 0.23 × 10-3 mm2/s, which is comparable with previous studies (Figure 2b). Plaque areas in DWI were compared with those in post contrast T1 weighted images and lipid-rich necrotic core findings were consistent (Figure 1b).

## Conclusions

Diffusion-prepared TSE allows, for the first time, 3D diffusion imaging of the carotid arterial wall in vivo with high spatial resolution and excellent image quality. It has the potential to detect vulnerable carotid plaque components such as lipid-rich necrotic core in vivo without the use of the contrast media.

## Funding

NIH/NHLBI R01HL096119.
